# Clinical and laboratory profile of urban sporotrichosis in a tertiary hospital in the city of São Paulo^☆^^☆☆^

**DOI:** 10.1016/j.abd.2020.07.010

**Published:** 2021-02-05

**Authors:** John Verrinder Veasey, Milton Ferreira Neves Neto, Ligia Rangel Barbosa Ruiz, Clarisse Zaitz

**Affiliations:** Dermatology Clinic, Santa Casa de Misericórdia de São Paulo, SP, Brazil

Dear editor,

Sporotrichosis is the most frequent subcutaneous mycosis in Latin America, where it is considered endemic.^1^ At the end of the 20^th^ century, the first cases of zoonotic transmission were described in Rio de Janeiro, initiating an epidemic outbreak that extended to other regions of Brazil.^2^, ^3^, ^4^ The lack of notification of this disease hides its real scope in the country, a fact also observed in its most populous city, São Paulo.^5^, ^6^ This study presents clinical and laboratory data of sporotrichosis cases treated at a tertiary hospital in downtown São Paulo from 2012 to 2020, aiming to increase the knowledge of this disease. This was a retrospective study analyzing data from medical records of patients attended at the dermatology clinic of that hospital. In each case, patient characteristics (age, sex, comorbidities, and contact with a diseased animal), disease characteristics (location, time of disease, clinical form, and treatment), and diagnostic methods (direct mycological examination [DME], fungal culture [FC], and histopathological examination [HE]) were evaluated.

During the analyzed period, 20 patients were treated, with age ranging from 2 to 81 years (mean of 32.2 ± 25.10 years), 55% female and 45% male. As for zoonotic screening, 30% denied contact with an animal and 70% declared previous contact with a sick cat; no other animals were mentioned. The results obtained are described in Table 1.

**Table 1:** Description of the clinical and laboratory aspects of the cases attended at the infectious dermatosis sector of a hospital in the center of the city of São Paulo from January 2012 to April 2020.

Case	Age	Sex	Contact with sick animal	Injury location	Disease duration (weeks)	Clinical presentation
1	58	Female	No	Upper limb	20	Lymphocutaneous
2	16	Female	No	Trunk	22	Lymphocutaneous
3	45	Female	Yes (cat)	Face and upper and lower limbs	13	Multiple inoculation
4	20	Male	Yes (cat)	Upper limbs	8	Multiple inoculation
5	10	Female	No	Upper and lower limbs	9	Immunoreactive – nodular erythema
6	11	Female	Yes (cat)	Face	10	Lymphocutaneous
7	12	Male	No	Face, upper limb, and back	44	Multiple inoculation
8	81	Female	No	Upper limb	24	Lymphocutaneous
9	3	Male	Yes (cat)	Face	4	Ocular mucosa
10	46	Male	No	Upper limb	167	Fixed cutaneous
11	42	Female	Yes (cat)	Upper limb	9	Fixed cutaneous
12	72	Female	Yes (cat)	Upper limb	9	Lymphocutaneous
13	30	Female	Yes (cat)	Upper limb	9	Lymphocutaneous
14	10	Female	Yes (cat)	Upper limb	9	Lymphocutaneous
15	12	Male	Yes (cat)	Face	5	Lymphocutaneous + ocular mucosa
16	75	Male	Yes (cat)	Upper limbs	39	Multiple inoculation
17	2	Male	Yes (cat)	Face	4	Fixed cutaneous
18	39	Male	Yes (cat)	Upper limbs and back	3	Multiple inoculation
19	13	Male	Yes (cat)	Upper limb	5	Lymphocutaneous
20	47	Female	Yes (cat)	Upper limb	4	Lymphocutaneous

Regarding the characteristics of the disease, lesions were present from three to 167 weeks until the appointment with the dermatologist, with a mean of 20.85 ± 36.24 weeks. The limbs were the most affected sites, totaling 15 cases (75%), with two cases of upper and lower limb concomitance and two cases of limbs and face, which was the second most affected site (six patients; 30%). The lymphocutaneous form was the most frequent (50% of cases; 10 patients), followed by multiple-inoculation in five cases (25%), and fixed-cutaneous in three cases (15%); two patients presented the ocular-mucosal form and one, the immunoreactive (erythema nodosum; Fig. 1). It is interesting to note that the majority of cases (60%) were patients in the extremes of the age scale, corresponding to the population with greater contact with sick animals, a finding compatible with that described in the literature; furthermore, the fact that all patients with facial involvement were children indicates the close facial contact that patients of this age group maintain with the animal.^7^ The lymphocutaneous form is the main clinical manifestation of sporotrichosis, representing 80% of reported cases.^2^, ^4^ In the present study, this form was the main clinical manifestation; however, it was observed in only half of the registered cases. The other half of cases presented the other manifestations, indicating that presentations described as atypical may represent a greater proportion than expected. Another noteworthy fact was that the patients' comorbidities did not indicate states of intense immunosuppression, explaining the presence of exclusively cutaneous clinical forms, without any case of systemic involvement.

**Figure 1:**
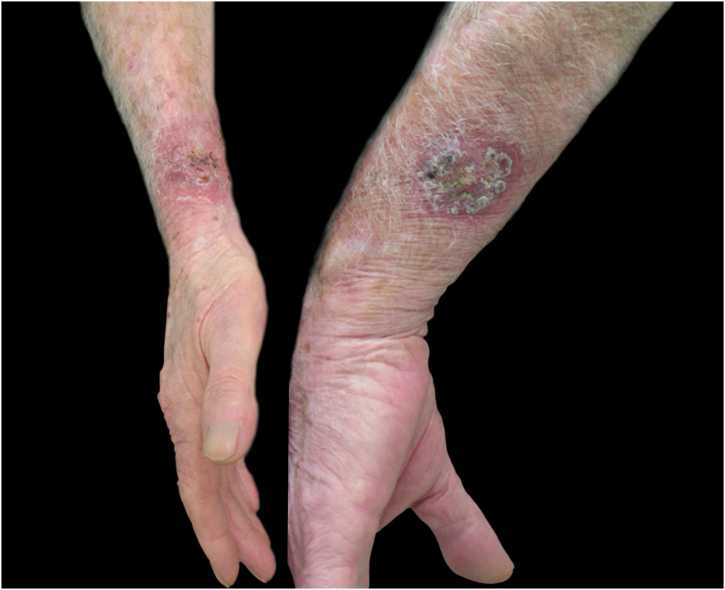
Sporotrichosis, cutaneous form of multiple inoculation in both forearms presented by case 16.

For diagnostic confirmation, lesion scrapings were performed in all cases for DME and FC analysis; in 16 cases, a biopsy was also performed for HE and FC analysis. Biopsy was not performed in four cases due to the refusal of patients or guardians. HE was analyzed with hematoxylin-eosin, Grocott-Gomori, and PAS stains; the fungus was detected in only five (31.25%), similarly to review studies that report positivity between 18% and 35.3% of the cases.^4^ The presence of yeasts was not found in any DME as expected, since the test has low sensitivity and specificity when compared with culture.^4^, ^8^ In the scraping and biopsy fragment cultures grown on Sabouraud and Mycosel agar, the fungus *Sporothrix* sp. was isolated in 100% of cases. Thus, it is confirmed that the gold standard method for the diagnosis of sporotrichosis is the culture and identification of species of the genus *Sporothrix* based on the material collected from skin lesions.^2^, ^4^, ^9^ In all cases presented here, the culture was identified phenotypically, by colonies with a membranous aspect and a white to beige bicolor with a blackish halo, at 25 °C. An analysis of the micromorphological aspect of the colonies at this same temperature was also carried out in all cases, showing hyaline septate hyphae with conidiophores, whose extremities presented piriform or rounded hyaline conidia in a “daisy” arrangement (Fig. 2).

**Figure 2:**
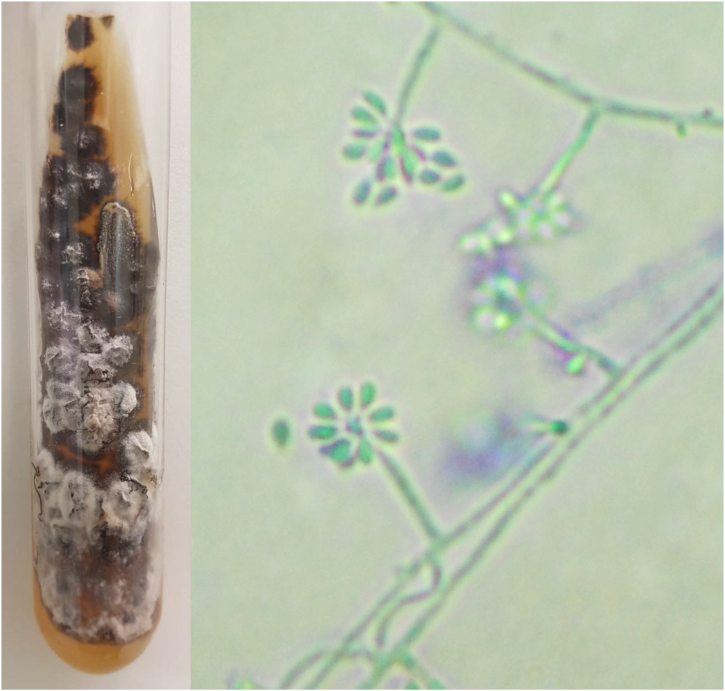
Phenotypic aspects of *Sporothrix* sp. Macromorphology on Mycosel agar medium at 25 °C with white to beige and black bicolor filamentous colony, and potato agar micromorphology (cotton blue, ×400) showing hyaline septate hyphae with conidiophores, with hyaline conidia at the extremities in a “daisy” arrangement.

All patients were treated with oral medication, with options of choice being a saturated solution of potassium iodide, itraconazole, and terbinafine, in single or associated use, with variable dose and duration in each case. These are all drugs indicated for the treatment of this infection for localized cases in immunocompetent patients; however, the fact that these medications were not supplied at the service where the appointments took place and the difficulty for patients to find the medication available for free in the public health network did not allow a standardization in order to present the findings in an organized manner.^2^, ^4^ However, in all cases, clinical cure was reached with the prescribed treatments.

In epidemiological terms, there were 19 autochthonous cases from the greater São Paulo area and one from the state of Bahia, with a progressive increase in incidence over the analyzed period. When screening for family cases, positivity was found in only three cases.

The present study highlights clinical and diagnostic characteristics of great importance, restating previous concepts and presenting ideas that should be analyzed in future similar research in the city in order to obtain greater knowledge and control of the disease. The present findings also highlight the need for stronger epidemiological surveillance, aiming to control the spread of this disease and prevent its progression, as occurs in other states and countries.^1^, ^7^, ^8^, ^9^, ^10^

## Financial support

None declared.

## Authors’ contributions

John Verrinder Veasey: Conception and planning of the study; elaboration and writing of the manuscript; obtaining, analyzing, and interpreting the data; effective participation in research orientation; intellectual participation in propaedeutic and/or therapeutic conduct of studied cases; critical review of the literature; critical review of the manuscript; approval of the final version of the manuscript.

Milton Ferreira Neves Neto: Elaboration and writing of the manuscript; obtaining, analyzing, and interpreting the data; approval of the final version of the manuscript.

Ligia Rangel Barbosa Ruiz: Effective participation in research orientation; intellectual participation in propaedeutic and/or therapeutic conduct of studied cases; critical review of the literature; critical review of the manuscript; approval of the final version of the manuscript.

Clarisse Zaitz: Effective participation in research orientation; intellectual participation in propaedeutic and/or therapeutic conduct of studied cases; critical review of the literature; critical review of the manuscript; approval of the final version of the manuscript.

## Conflicts of interest

None declared.
